# 90-Day Survival of Domestic Sheep (*Ovis aries*) in a Rock Trap: The Case “Monyak”

**DOI:** 10.3390/ani16142198

**Published:** 2026-07-15

**Authors:** Rusko Petrov, Mirela Kazakova, Mehmed Halil

**Affiliations:** 1 Section Ecology and Zoology, Department of General Animal Husbandry, Faculty of Veterinary Medicine, Trakia University—Stara Zagora, 6000 Stara Zagora, Bulgaria; rpetrov@greenbalkans.org; 2 Green Balkans—Stara Zagora NGO, 26 Konstantin Irechek Str, 6000 Stara Zagora, Bulgaria; 3 Section Cytology, Histology and Embryology, Department of Veterinary Anatomy, Histology and Embryology, Faculty of Veterinary Medicine, Trakia University—Stara Zagora, 6000 Stara Zagora, Bulgaria; 4 Section Veterinary Hygiene, Ethology and Animal Welfare, Department of General Animal Husbandry, Faculty of Veterinary Medicine, Trakia University—Stara Zagora, 6000 Stara Zagora, Bulgaria; mehmed.halil@trakia-uni.bg

**Keywords:** *Ovis aries*, sheep, extreme environmental conditions, prolonged isolation, starvation, dehydration, adaptive responses, stress physiology, maternal behavior, veterinary clinical pathology

## Abstract

The present study describes a rare and extreme case of prolonged survival in domestic sheep (*Ovis aries*) under severe natural conditions, designated as the “Monyak” case (March 2026, Eastern Rhodope Mountains, Bulgaria). A 90-day period of complete isolation involving two adult ewes and a subsequently born lamb on an inaccessible rocky ledge, with very limited access to sources of food or water, was documented. Clinical assessment performed immediately following rescue revealed profound dehydration and cachexia accompanied by multisystemic abnormalities suggestive of a prolonged catabolic state. Biochemical analysis demonstrated marked azotemia, characterized by elevated serum urea and creatinine concentrations, which may indicate reduced renal perfusion and intensified protein catabolism. Electrolyte disturbances, including hypernatremia and hyperchloremia, were compatible with severe dehydration and substantial disruption of water−electrolyte homeostasis. Furthermore, increased total serum protein concentration and hematocrit values were observed, consistent with pronounced hemoconcentration. The metabolic profile demonstrated alterations in glucose homeostasis together with enhanced ketogenesis, findings compatible with an adaptive metabolic response to sustained energy deprivation. Elevated serum activities of aspartate aminotransferase (AST) and alanine aminotransferase (ALT), accompanied by increased cortisol concentrations, may reflect hepatocellular stress and activation of the hypothalamic–pituitary–adrenal (HPA) axis associated with chronic physiological stress. Hematological evaluation revealed neutrophilia and lymphopenia, constituting a leukocyte pattern compatible with a stress leukogram and suggestive of endogenous glucocorticoid stimulation. Morphological examination of peripheral blood smears demonstrated pronounced echinocytosis and increased relative cellular density secondary to plasma volume depletion associated with dehydration. Additionally, neutrophilic hypersegmentation was observed, consistent with chronic physiological stress and cellular exhaustion. Ethological assessment suggests that maternal behavior and instinctive maternal care played a pivotal role in ensuring neonatal survival under conditions of extreme resource scarcity and sustained environmental stress. In conclusion, the “Monyak” case provides a unique natural model for investigating the physiological, metabolic, and behavioral adaptive mechanisms of ruminants subjected to prolonged starvation, dehydration, and chronic stress. The findings contribute valuable insights to veterinary physiology, clinical pathophysiology, and ethology, with potential implications for understanding the limits of adaptive capacity and survival under extreme environmental conditions.

## Simple Summary

This study describes a rare case of prolonged survival in domestic sheep isolated for approximately 90 days on an inaccessible rocky ledge in the Eastern Rhodope Mountains, Bulgaria. Two adult ewes and a lamb born during the isolation period survived with very limited access to food and water. After rescue, the animals showed severe dehydration, loss of body condition, and physiological changes associated with prolonged energy deprivation and chronic stress. Despite these extreme conditions, maternal behavior and nursing activity were preserved, which may have supported lamb survival. The “Monyak” case provides a unique opportunity to investigate the adaptive responses of ruminants to prolonged nutritional and environmental stress and contributes to veterinary physiology, clinical pathology, and animal welfare research.

## 1. Introduction

Domestic sheep possess a long history of adaptation to diverse and frequently harsh grazing environments in Bulgaria [1,2]. Traditionally, sheep husbandry has been practiced in mountainous and semi-mountainous regions such as the Rhodope Mountains, Balkan Mountains, and Strandzha Mountains, where extensive pastoral systems enabled seasonal livestock movements (transhumance) and the utilization of nutritionally limited yet ecologically diverse pasture resources [3,4]. Within this context, particular attention should be given to local Rhodope autochthonous and primitive sheep populations, which historically underwent natural and husbandry-driven selection with minimal influence from modern breeding genetics [1]. These animals are characterized by considerable resilience to climatic fluctuations, efficient utilization of low-quality grazing resources, and pronounced adaptation to steep and highly fragmented mountainous landscapes.

Among the sheep populations most closely associated with the region are those related to the Karakachan sheep, Rhodope sheep, and other indigenous local types traditionally raised in the Rhodope Mountains under extensive grazing systems. These animals exhibit substantial behavioral plasticity, a strong flocking instinct, and an exceptional ability to navigate inaccessible and rugged mountainous terrain, making them particularly well adapted to the environmental conditions of the Eastern Rhodopes [5,6].

The Eastern Rhodope Mountains, and more specifically the “Monyak” Fortress area located in close proximity to the village of Shiroko Pole, Kardzhali Municipality, Bulgaria, are characterized by highly complex geomorphology, including steep rock formations, karst features, and near-vertical slopes with local elevation differences reaching approximately 200–300 m in certain sections of the terrain. The “Monyak” Fortress itself is situated atop a rocky peak at an elevation of approximately 590 m above sea level. The fortress occupies a relatively small area on a narrow and difficult-to-access rocky terrace dominated by steep cliff faces.

The incident began more than two months prior to the rescue operation when a flock of domestic sheep belonging to the livestock farmer Hilmi Hayrula was startled by a tourist’s dog while the tourist was ascending toward the summit of the “Monyak” Fortress. As a consequence of the ensuing panic response, the animals moved toward the edge of the rocky escarpment. Three sheep fell to their deaths, whereas two adult ewes survived by landing on an extremely inaccessible rocky ledge with an estimated surface area of approximately 350 m^2^. This ledge is surrounded by steep cliffs exceeding 20 m in height in several sections and by substantially higher rock faces within the broader geomorphological context of the area.

**Figure 1:**
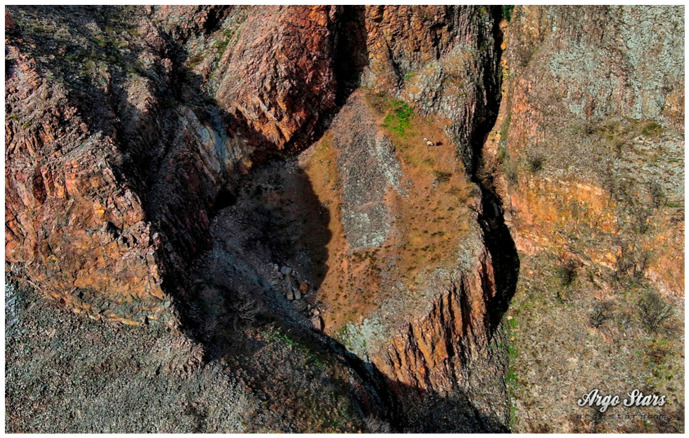
Picture: the rocky ledge; Georgi Argirov-Argo.

For nearly 90 days, the animals remained completely isolated without direct access to conventional sources of forage and water (Figure 1). During this prolonged period, an additional complication emerged approximately one month after the initial incident when one of the ewes successfully gave birth to a live and viable lamb. This event represents a particularly remarkable biological phenomenon given the extreme circumstances of prolonged restriction of food and water availability, combined with chronic stress. The survival of the newborn lamb immediately after parturition further emphasizes the extraordinary physiological resilience and maternal capabilities of domestic sheep under severe environmental constraints.

## 2. Materials and Methods

The study comprised a clinical, laboratory, and ethological investigation of one adult domestic ewe (*Ovis aries*) and its neonatal lamb, rescued from an inaccessible rocky ledge in the vicinity of the Monyak Fortress, Eastern Rhodope Mountains, Bulgaria. Laboratory investigations, including biochemical, hematological, and morphological analyses, were performed exclusively on the adult ewe (*n* = 1), from which a venous blood sample was collected. The lamb underwent only a comprehensive clinical examination and post-rescue ethological observation. The third adult ewe, located on the same rocky ledge, could not be rescued and was therefore excluded from the study.

Following the rescue operation, both surviving animals underwent a complete clinical examination and assessment of body condition. Venous blood was collected exclusively from the adult ewe (*n* = 1) for serum biochemical analysis, hematological evaluation, preparation of peripheral blood smears, and cytomorphological examination. The obtained laboratory data were interpreted by comparison with established reference intervals for clinically healthy adult sheep.

In addition, post-rescue ethological observations were conducted to assess behavioral responses, maternal interactions, and adaptive behavioral patterns exhibited by both animals during the recovery period.

The investigated animals consisted of one adult female domestic sheep (*Ovis aries*) and its lamb. The adult ewe was approximately four years of age and had a lactational reproductive status following parturition under conditions of prolonged isolation. Based on morphological assessment and information provided by the owner, the animal was identified as a local crossbreed exhibiting predominant phenotypic characteristics of the Karakachan sheep breed.

The ewe’s pre-incident body weight was estimated visually by the owner and the rescue team at approximately 50 kg. Following the rescue, body weight was estimated at approximately 35 kg using visual assessment and palpation, as accurate weighing was not feasible under field conditions.

Body condition was assessed using the 5-point Body Condition Score (BCS) system, which is widely applied in small ruminants. Scoring was performed independently by three experienced observers, and the final BCS was established by consensus to minimize subjective variability. Prior to the isolation period, the ewe’s BCS was estimated at 3.5/5, whereas post-rescue evaluation yielded a BCS of 1.5/5, indicative of severe cachexia and marked depletion of adipose energy reserves.

The blood sample was obtained by jugular venipuncture (*Vena jugularis*) and placed in a sterile vacuum tube without anticoagulant, intended for serum collection.

Serum biochemical parameters were determined using an automated clinical chemistry analyzer based on photometric (wet chemistry) methodology, in accordance with the manufacturer’s calibration procedures and internal quality control protocols.

Hematological parameters were analyzed using an automated veterinary hematology analyzer employing electrical impedance technology in combination with flow cytometry.

The laboratory parameters were interpreted by comparing control reference values (healthy animals) with the values recorded in the rescued animals from the case “Monyak” [7,8]. The reference intervals applied in this study are derived from multiple ovine populations and are not breed-specific.

For the preparation of blood smears, venous blood was collected from the evacuated animals into sterile vacuum tubes containing an anticoagulant. Under field conditions, air-dried blood samples placed on microscope slides were stained using the Hemacolor Rapid Staining of Blood Smears kit (Manufactured by Merck KGaA, Darmstadt, Germany), which includes methanol as a fixative, Reagent 2 and Reagent 3 as staining solutions, and buffer solution at pH 7.2 [9]. The stained smears were examined and evaluated using a Levenhuk MED DN-200M/VDN-200M microscope (Levenhuk, Inc., Tampa, FL, USA), at ×1000 magnification using a semiquantitative assessment approach.

All laboratory analyses were conducted at the Diagnostic Laboratory of the University Veterinary Teaching Hospital, Trakia University, operating in accordance with standardized protocols for routine veterinary diagnostics and an established internal quality assurance system.

Due to the constraints of field conditions and the retrospective nature of the observations, individual laboratory results were not available for statistical analysis. The study is based on a single clinical case (*n* = 1); therefore, the findings are presented in a descriptive manner through comparison with published reference intervals for clinically healthy sheep.

Prior to the final rescue mission, two unsuccessful evacuation attempts were recorded, carried out by local services (fire brigade and civil protection), limited by the difficult terrain, the friable rocky substance, and the high risk of inducing panic in the animals. Due to the complexity of the situation, a decision was made to apply a combined approach, including controlled sedation and alpine rescue techniques. The adult ewe was sedated via intravenous administration (*Vena jugularis*) of Neurotranq^®^ (acepromazine maleate 10 mg/mL) (Alfasan International B.V., Woerden, The Netherlands) in order to minimize stress response and ensure safe handling during the rescue operation. Body weight was estimated visually and by palpation at approximately 35 kg.

Acepromazine was administered at a dose of 0.02–0.05 mg/kg, adjusted according to the animal’s overall clinical condition, degree of exhaustion, and the requirement to achieve mild sedation without induction of loss of consciousness. The selection of acepromazine was based on its moderate sedative properties, its ability to reduce reactivity to external stimuli, and its established applicability for short-term restraint procedures in ruminants.

The achieved level of sedation was considered adequate for safe capture, placement into a transport harness, and execution of vertical extraction, without progression to deep sedation or general anesthesia.

On 17 March 2026, following an approximately 90-day period of isolation for the animals, a multidisciplinary team of around 20 people was deployed in the field after mobilization from the city of Stara Zagora. The team comprised specialists from the Wildlife Rescue Center of “Green Balkans” (Stara Zagora), as well as students from the Faculty of Veterinary Medicine at Trakia University. The operation was organized as a coordinated field intervention in a high-risk mountainous environment characterized by restricted access and significant vertical relief. The entire incident period spanned the winter season of December 2025−March 2026, marked by low temperatures, challenging terrain conditions, and severely limited accessibility of the area.

Throughout the isolation period, local residents and the animals’ owner made repeated attempts to provide supplementary feeding and water by throwing food from accessible vantage points. However, these attempts proved unsuccessful due to the presence of steep vertical rock faces, which prevented the materials from reaching the rocky terrace where the animals were located.

The rescue strategy was implemented through the functional division of the team into several operational units with clearly defined responsibilities. The Alpine team was responsible for direct access to the two rescued animals (one adult ewe and its neonatal lamb), the execution of controlled capture procedures, the administration of sedation in the adult ewe, and the placement of both animals into specialized transport harnesses. These operations were carried out while traversing vertical terrain with an elevation gain exceeding 300 m. The operational−technical team managed rope systems, ensured safety control during vertical hauling procedures, and maintained continuous communication with the alpine team throughout the operation.

The logistics team provided support for the transport of heavy alpine equipment in conditions of no vehicle access, organized the upper operational base, and received the animals after extraction from the rocky ledge [10]. The detective−observational team was positioned beneath the rock formation, performing continuous visual monitoring, real-time risk assessment, and coordination of inter-team movements [11].

During the operation, a rotational transport team was also formed, functioning dynamically according to terrain demands. This unit carried out a circumferential route around the rock massif, followed by an ascent phase and final transport of the animals to the nearest settlement. The entire operation was conducted under conditions of high risk, limited visibility, and severely restricted terrain accessibility, requiring tightly coordinated actions among all participating units.

**Figure 2:**
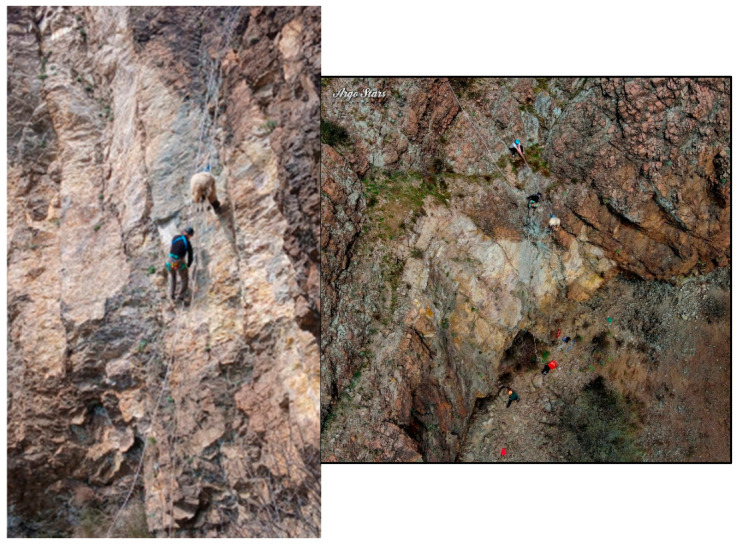
Pictures: Evacuation of the ewe; Rusko Petrov and Georgi Argirov-Argo.

Specialized alpine and rock-climbing equipment was employed, including rope-system technology and radio communication devices enabling coordination among all team members (Figure 2). Nearly all participants had undergone specialized training and practical exercises for similar high-risk rescue operations as part of their academic preparation at the university.

The rescue team consisted of specialists from the Wildlife and Domestic Animal Rescue Unit, an organization established in 2013 by Dr. Rusko Petrov. The rescue operation was personally directed by Dr. Petrov and supported by trained students from the Faculty of Veterinary Medicine at Trakia University, Stara Zagora, Bulgaria.

## 3. Results

**Figure 3:**
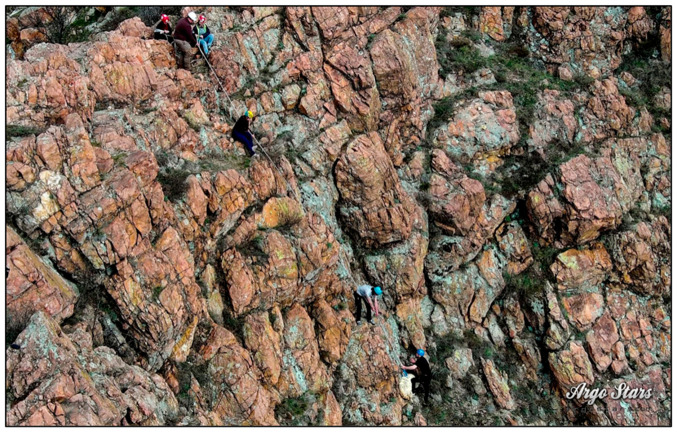
Picture: Multi-stage rope-assisted evacuation of the ewe; Georgi Argirov-Argo.

The evacuation of the ewe and the lamb required approximately 14 h of intensive rescue operations under extremely challenging terrain conditions (Figure 3). During the operation, an additional incidental behavioral event was observed in the third isolated animal, which attempted to escape and subsequently fell onto a lower rocky terrace, further confirming the high instability of the terrain and the substantial risk associated with the rescue operation.

All clinical and laboratory analyses pertain to a single investigated subject (*n* = 1), representing the adult ewe, whereas the lamb was included exclusively in clinical and ethological observation.

Immediate post-evacuation clinical examination showed severe dehydration and pronounced cachexia, with body weights in the adult animals recorded below 35 kg. Despite severe cachexia and dehydration, both rescued animals were alive and clinically stabilized immediately following evacuation, with no evidence of acute fatal outcome or indication for euthanasia. Marked muscular atrophy was observed; however, vital signs and responsiveness remained preserved. In the ewe, markedly reduced lactation was documented, which nevertheless remained functionally sufficient to ensure the survival of the newborn lamb, representing an important adaptive and ethological outcome of the case.

Biochemical laboratory analyses demonstrated marked deviations from reference values established for healthy sheep (Table 1). A substantial increase in blood urea concentrations (18.4 mmol/L) was recorded, which can be indicative of severe dehydration and catabolic metabolism. Elevated creatinine levels (312 µmol/L) were also observed, suggestive of renal stress and reduced tissue perfusion. Hypernatremia (168 mmol/L) and hyperchloremia (128 mmol/L) were present, findings indicating possible electrolyte imbalance. Total serum protein (92 g/L) and hematocrit values (58%) were increased, which could be associated with hemoconcentration secondary to dehydration. Blood glucose levels were reduced (1.9 mmol/L), consistent with prolonged starvation and hypoglycemia, while concurrently elevated ketone bodies are suggestive of ketosis. Hepatic enzymes AST (286 U/L) and ALT (78 U/L) were increased, changes that may reflect hepatocellular and muscular stress, while cortisol concentrations (245 nmol/L) were significantly elevated as a marker of chronic systemic stress [12,13].

Due to the single-sample nature of the investigation (*n* = 1), the results are descriptive and comparative in character and do not permit statistical analysis or the establishment of population-level associations.

Hematological and morphological examination of peripheral blood smears revealed relative erythrocytosis secondary to hemoconcentration, the presence of echinocytes as an indicator of electrolyte imbalance, and a typical stress leukogram characterized by neutrophilia (70–85%) and lymphopenia (10–25%). Additionally, neutrophil nuclear hypersegmentation was observed, representing a morphological sign of chronic stress and physiological exhaustion (Table 2) [14,15].

Ethological observations suggested that, despite prolonged isolation and severe resource limitation, the ewe retained maternal behavior and exhibited adaptive behavioral responses throughout the observation period. The continued presence of lactational activity, together with the survival of the lamb, may indicate that maternal investment was maintained at a level sufficient to support offspring viability under these conditions, although the extent of milk production could not be quantified. Furthermore, a high risk of secondary incidents was recorded, including the fall of one animal onto a lower terrace due to acute stress during the rescue operation, confirming the extreme nature of the environmental conditions.

Field observations conducted immediately following evacuation documented preserved maternal behavior toward the neonatal lamb, including continued caregiving and nursing behavior despite severely restricted resource availability. These observations are consistent with the persistence of maternal care under conditions of prolonged physiological stress.

## 4. Discussion

During the rescue operation, the ewe displayed an unexpected calm behavioral state and relative stability. While domestic sheep (*Ovis aries*) typically exhibit pronounced panic responses and disorganized escape behavior under comparable conditions, the observed case differed in that the animal did not show overt panic or marked flight reactions. This behavior may have been influenced by a strongly expressed maternal motivation, which modulated the typical “fight-or-flight” response [16].

A particularly significant aspect of the case is that the ewe gave birth to a live and clinically healthy lamb during the period of isolation. This event appears to have been an important concurrent factor in the course of the incident. The presence of the newborn may have contributed to the maintenance of maternal motivation and behavioral focus, potentially influencing the overall stability of the animal. In addition, a limited but sustained lactation capacity may have supported survival during the isolation period.

This context positions the “Monyak” case as an exceptionally atypical example of prolonged survival under extreme rocky isolation, combining elements of behavioral adaptation, physiological stress, and specific geographic constraints. Domestic sheep (*Ovis aries*) are highly social animals, for which prolonged isolation is associated with elevated stress hormone levels, including cortisol, as well as behavioral and physiological changes resulting from both social and nutritional deprivation [17]. Nevertheless, the observed combination of maternal behavior, partially preserved physiological function, and extreme adaptation to minimal resources renders this case a valuable model for understanding the limits of physiological and behavioral resilience in ruminants.

Sheep adapted to arid and semi-arid environments possess morphological adaptations necessary for migration across mountainous terrain where nutritional resources are fragmented and scarce. They are characterized by relatively long limbs and a body conformation optimized for thermoregulation and minimal water loss, contributing to integrated physiological efficiency [18]. These breeds are well known for their resilience under harsh environmental conditions, abrupt climatic fluctuations, and limited water availability. Under water deprivation, a cascade of physiological changes occurs, including reduced feed intake and body weight loss, production of small volumes of highly concentrated urine, hemoconcentration, and immunosuppression [19]. Other authors [20] have reported that in sheep adapted to arid regions, water restriction does not significantly affect body mass or wool quality. The “Monyak” case suggests that local sheep breeds may possess adaptive mechanisms that could contribute to resilience under severe environmental stress, as illustrated by the animal’s survival during the 90-day period of isolation on the rocky terrace.

Compared with large ruminants, small ruminants demonstrate higher survival rates under conditions of water scarcity [21].

Due to the social nature of sheep, isolation constitutes a major stressor. Separation from the flock adversely affects both individual welfare and overall flock cohesion [22]. A key factor contributing to survival at the “Monyak” Fortress was the persistence of sociality under constrained conditions. The two adult individuals and the newborn lamb adapted to a form of isolated yet socially structured existence, effectively maintaining a collective survival unit.

Reproductive status is closely associated with stress levels. Some authors [23] reported increased sensitivity to external stimuli and social isolation in female sheep during estrus. Conversely, stress levels shift around parturition. Naturally, prepartum behavior includes maternal withdrawal from the flock. In ewes, the lowest stress responses to social isolation are recorded during the periparturient period, while hormonal profiles in late pregnancy significantly suppress stress reactivity [24]. Despite the extreme conditions on the rocky terrace, maternal instinct in the pregnant ewe before and after parturition constituted a key survival factor.

A newborn lamb possesses limited energy reserves and requires rapid access to colostrum to maintain thermoregulation and survival. A critical component of the imprinting period is the location of birth [25]. Contrary to expectations, the restricted access to food, near absence of water, and harsh rocky environment in the “Monyak” case further strengthened the maternal−offspring bond, as also evidenced during the rescue operation.

Physiological stress during lactation, induced by metabolic strain, is directly associated with elevated cortisol levels. Cortisol concentrations increase significantly above baseline following stress-inducing events in lactating ewes [26]. Biochemical analysis of blood samples from the rescued ewe similarly revealed elevated cortisol levels accompanied by increased hepatic enzyme activity (AST, ALT).

Elevated serum cortisol is associated with reduced milk production. Maternal physiological and physical distress disrupts oxytocin secretion, thereby reducing milk output [27]. Conversely, other authors [28] reported that in the early postpartum period, oxytocin release occurs in pulsatile patterns, and the frequency of oxytocin pulses during early nursing is associated with higher milk yield and prolonged lactation. Severe stress conditions around parturition directly inhibit the neuroendocrine mechanisms required for the initiation and maintenance of lactation. Following the rescue operation, absence of lactation was recorded in the ewe. Nevertheless, the survival of the lamb indicates that, despite extreme environmental conditions and severe maternal distress, sufficient milk secretion had been available to ensure early postnatal survival.

Studies on emaciated, malnourished, and growth-retarded small and large ruminants show significantly reduced erythrocyte count, hemoglobin concentration, and hematocrit compared with healthy animals. The change in those parameters is indicative of anemia [29]. Analyses of the rescued animals from the “Monyak” Fortress demonstrated hemoconcentration with elevated hematocrit values and no sign of anemia.

Prolonged malnutrition induces leukocytic alterations resulting in immune system impairment. Neutrophilic changes and lymphocytopenia are reliable indicators of immune dysfunction [30]. This was also demonstrated in the morphological analysis of blood smears from the evacuated animals, which revealed neutrophilia with nuclear segmentation and lymphocytopenia, characteristic of a classical stress leukogram.

One of the most sensitive indicators of metabolic decompensation in sheep subjected to prolonged starvation is blood glucose concentration. Glucose levels may decrease below 2.8 mmol/L as a result of depletion of hepatic glycogen reserves [31]. The biochemical results from the rescued animals confirmed this pattern, with glucose values ranging between 1.5 and 2.0 mmol/L.

Elevated blood urea levels represent one of the earliest and most consistent findings in metabolic stress resulting from prolonged starvation in sheep. Serum urea increases significantly compared with healthy animals, reaching 2- to 5-fold above normal values [32]. The same authors also reported creatinine dynamics in malnourished and dehydrated animals. Creatinine is a marker of glomerular filtration rate and renal clearance. In dehydration, plasma creatinine increases, whereas muscle loss during starvation reduces its production, potentially resulting in paradoxically normal creatinine values in severely malnourished and dehydrated animals.

## 5. Conclusions

The “Monyak” case represents a rarely documented example of prolonged survival in domestic sheep (*Ovis aries*) under extreme field conditions characterized by prolonged isolation, restricted access to resources, and substantial physiological stress. The observed survival of two adult animals and a neonatal lamb over an approximately 90-day period under conditions of severely limited access to food and water reflects an extreme level of challenge to homeostatic mechanisms, without allowing generalizations regarding the species’ typical adaptive capacity.

The available data from this single case demonstrate pronounced signs of severe catabolic metabolism, dehydration, and systemic stress. Their interpretation should be considered within the context of an acute clinical presentation under extreme environmental conditions, rather than as a model of physiological adaptation or resilience. Due to limitations associated with the small number of animals and the single-case design, the findings cannot be used to derive broadly applicable conclusions regarding adaptive mechanisms in ruminants.

Behavioral observations, including maternal behavior and neonatal care, may be regarded as concomitant factors during the course of the event; however, the available data do not allow causal interpretation of their role in the outcome. Further controlled studies would be required to evaluate the influence of ethological factors under comparable extreme conditions.

In conclusion, the “Monyak” case is primarily of value as a descriptive clinical and ethological report, illustrating the spectrum of physiological alterations associated with severe and prolonged stress, but does not provide a basis for generalization regarding species adaptation or resilience.

## Figures and Tables

**Table 1:** Comparison of biochemical parameters (healthy animals vs. the case “Monyak”).

Parameter	Reference Range	The Case “Monyak”	Interpretation
Urea (mmol/L)	2.6–7.1	↑ 18.4	Severe azotemia/catabolism
Creatinine (µmol/L)	106–168	↑ 312	Prerenal hypoperfusion
Sodium (Na^+^ mmol/L)	140–155	↑ 168	Hypernatremia/dehydration
Chlorides (Cl^−^ mmol/L)	95–110	↑ 128	Electrolyte imbalance
Total protein (g/L)	60–79	↑ 92	Hemoconcentration
Hematocrit (%)	27–45	↑ 58	Severe dehydration
Glucose (mmol/L)	2.8–4.4	↓ 1.9	Negative energy balance
Ketone bodies	negative	↑ 3.2 mmol/L	Ketosis
AST (U/L)	40–123	↑ 286	Hepatic/muscle stress
ALT (U/L)	15–34	↑ 78	Hepatocellular stress
Cortisol (nmol/L)	42–82	↑ 245	Chronic stress response

Note: ↑ Elevated values; ↓ Reduced values.

**Table 2:** Comparison of hematological and morphological findings.

Parameter	Reference Range	The Case “Monyak”	Interpretation
RBC (×10^12^/L)	9–15	18.7	Relative erythrocytosis
Echinocytes	rare	frequent	Electrolyte imbalance
WBC (×10^9^/L)	4–12	14.9	Stress leukogram
Neutrophils (%)	40–60	82	Neutrophilia
Lymphocytes (%)	40–70	14	Lymphopenia
Neutrophil morphology	Normal	Hypersegmentation	Chronic stress/exhaustion

## Data Availability

The original contributions presented in this study are included in the article. Further inquiries can be directed to the corresponding author.
